# A facile and general approach for production of nanoscrolls with high-yield from two-dimensional nanosheets

**DOI:** 10.1038/s41598-018-33709-z

**Published:** 2018-10-15

**Authors:** Wucong Wang, Yanzhe Gai, Ding Xiao, Yaping Zhao

**Affiliations:** School of Chemistry and Chemical Engineering, Shanghai Jiao Tong University, Shanghai, 200240 P. R. China

## Abstract

Nanoscrolls (NSs) assembled from two-dimensional nanosheets have emerged as a novel type of one-dimensional nanomaterials because of their unique topological features and properties. The scale-up preparation of the NSs is crucial for their foundational and applied research. Herein, we report a general and straightforward approach for efficiently converting two-dimensional nanosheets into the NSs with high yield. We demonstrated the converting process by illustrating the formation of the graphene nanoscrolls through characterizing their morphology and structure using a scanning electron microscope, transmission electron microscope, Raman spectra, and X-ray diffraction spectra. The graphene sheets with a few-lay number were converted immediately and entirely into the graphene nanoscrolls when they mixed with an ethanol solution of silver nitrate at room temperature. The as-prepared graphene nanoscrolls were confirmed to be formed via the layer-by-layer assembly of graphene triggered by silver cyanide formed in site. Also, we extended this approach to construct the nanoscrolls of the hexagonal boron nitride, molybdenum disulfide, and tungsten disulfide, respectively, from their corresponding two-dimensional nanomaterials. In a broader context, this approach paves a significant new way for the large production of the NSs with cost-efficiency.

## Introduction

Carbon nanoscrolls were discovered when making multiwalled carbon nanotubes^1,2^. Since the discovery of graphene, the carbon nanoscrolls was convinced of deriving from scrolling of graphene, named as graphene nanoscrolls (denoted GNSs)^3,4^. GNSs inherit not only excellent properties of graphene, such as high mechanical strength, outstanding electrical conductivity, and superior carrier mobility but also possess open topological structure at both ends and interlayer galleries^5^. It endows the GNSs with wonderful features, such as excellent electronic and electromechanical properties^6,7^. Thus, the GNSs have significant potential applications in energy storages, photovoltaic cells and sensors^4,8–10^. Besides graphene, recently it was reported that the other layered two-dimensional nanomaterials (denoted 2DNMs) could be rolled into the nanoscrolls (denoted NSs) too, such as h-boron nitride (h-BN), tungsten disulfide (WS_2_), and molybdenum disulfide (MoS_2_)^11^. The NSs have become a new type of one-dimension nanomaterials with novel properties. Therefore, the preparation of the NSs has attracted increasing attention of scientific researchers. However, most of the research work is limited to theoretical prediction and calculation^12–15^. Only a few previously published articles involved fabricating the NSs. Moreover, the reported processes are complicated, and the conversion yield is tiny limiting their application^16–23^. Up to date, there is no general and efficient method to enable assembling the NSs with high-yield.

Herein, we report a facile and general approach for production of high-quality NSs with high-yield from two-dimensional nanosheets. The NSs are fabricated via self-scrolling of the 2DNMs in the solution of silver nitrate and ethanol. We have demonstrated the formation of the nanoscrolls of graphene, h-BN, MoS_2_, and WS_2_ by verifying the morphology and structure of the formed NSs using scanning electron microscope, transmission electron microscope, Raman spectra, and X-ray diffraction spectra. Also, the mechanism of the self-assembly scrolling processes is elucidated.

## Results and Discussion

**Figure 1:**
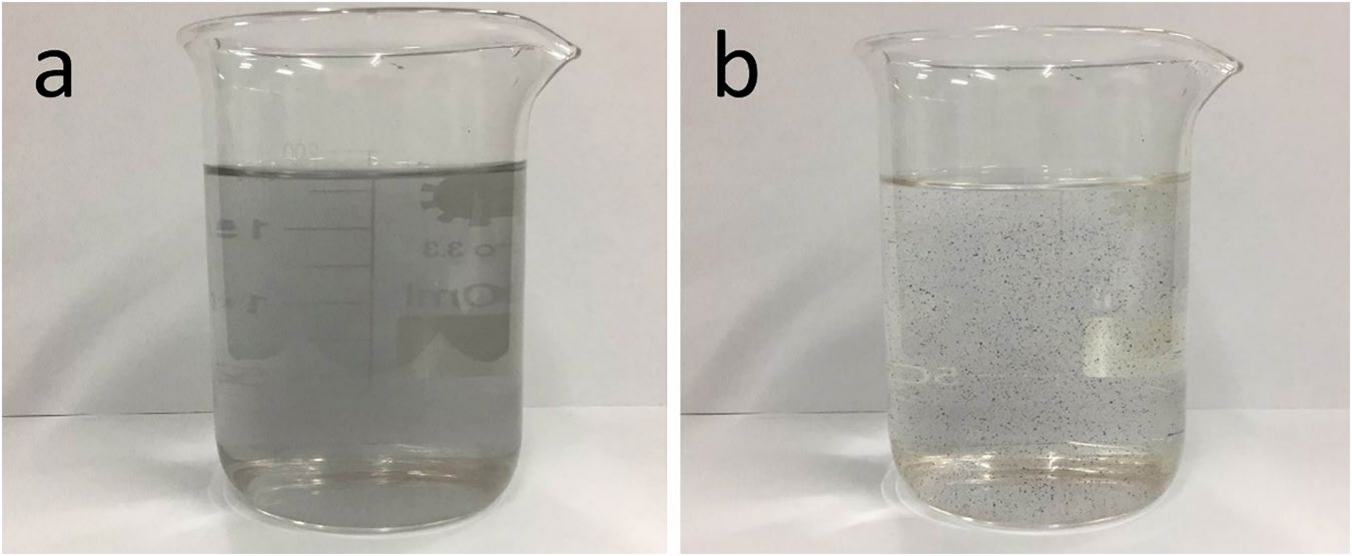
Digital photograph of (**a**) the dispersion of graphene in ethanol, and (**b**) the graphene nanoscrolls formed in ethanol solution.

The graphene sheets exfoliated from graphite are taken as the representative of the 2DNMs to discuss in detail. The profiles of the exfoliated graphene sheets are shown in Fig. S1, which indicates that the graphene is less than five layers with the lateral size of 1–3 µm. The appearance change of the reaction solution during the conversion of the graphene to the GNSs was recorded by a digital camera. Figure 1a displayed the initial state of the grey dispersion of the graphene and AgNO_3_ ethanol solution when they were mixed and magnetically stirred. After stirring the solution for some time, the grey dispersion gradually changed into a clear solution (Fig. 1b), from which it can be seen that a kind of fluffy particles was formed and deposited. These particles are confirmed to be the GNSs by a series of characterization methods.

**Figure 2:**
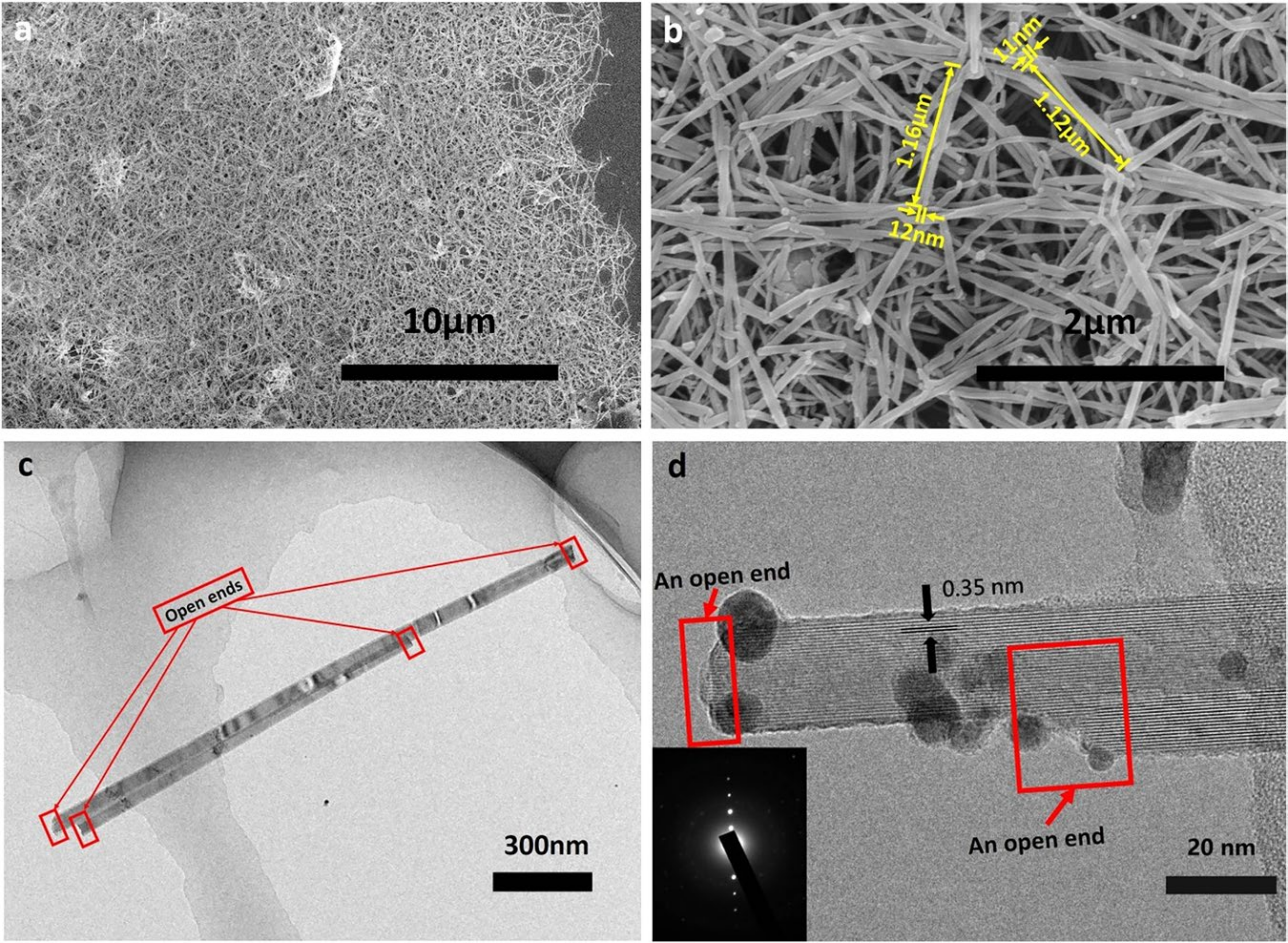
Microstructure and morphology of the GNSs. (**a,b**) SEM images of GNSs under different magnifications. (**c**) TEM image of two abreast GNSs. (**d**) High-magnification TEM image of the GNS and its typical electron diffraction pattern.

A scanning electron microscope (SEM) and a transmission electron microscope (TEM) were applied to characterize the microstructure and morphology of the GNSs. The SEM image (Fig. 2a) indicates that a mass of the GNSs was formed and the graphene sheets disappeared. It means that all of the exfoliated graphene sheets have been converted into the GNSs. It can be seen from the high-magnification SEM (Fig. 2b) that the GNSs uniformly scattered on the substrate and the length and diameter of the GNSs are in the range of 0.5–10 µm and 10–50 nm, respectively, which relates to the size of the original graphene sheets and the curling mode. The TEM images illustrate further the microstructure of the GNSs. Two abreast GNSs are shown in Fig. 2c. Their ends are open, and their lengths and diameters are 1.2–1.8 µm and 20–30 nm, respectively. The sizes of the GNSs measured from the TEM are consistent with ones from the SEM. The high-magnification TEM (Fig. 2d) displays that the interlayer spacing of the GNSs is about 0.35 nm which is as similar as the (002) distance of graphite. It suggests that the GNSs were scrolled up via layer by layer. Also, the typical electron diffraction pattern (inset of Fig. 2d) indicates the specific structure of the GNSs has an identical structure to the multiwalled carbon nanotubes^24^. However, the ends of the GNSs are open not like the closed structure of the multiwalled carbon nanotubes as shown in the red rectangles of Fig. 2d.

**Figure 3:**
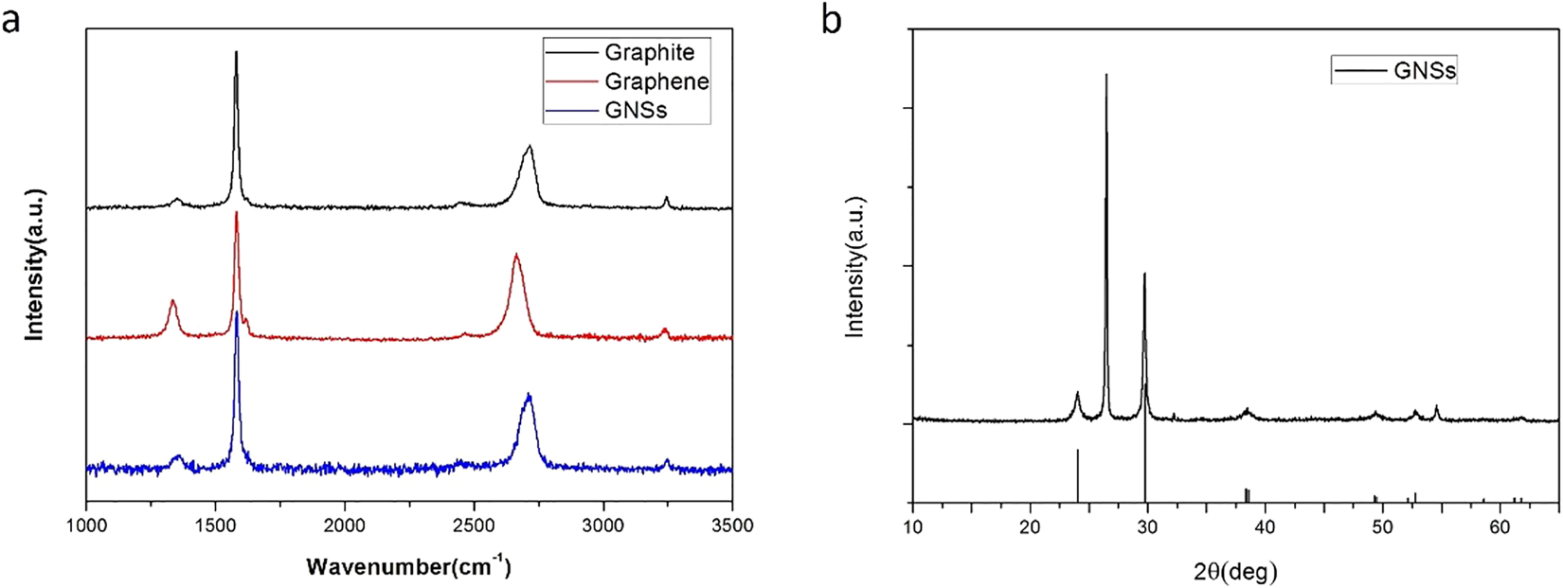
Raman and XRD characterization. (**a**) Raman spectrum of graphite, graphene and GNSs, (**b**) XRD patterns of GNSs and PDF card of AgCN (PDF#23-1404).

Raman spectrum is an excellent way to characterize the structure of the nanostructured materials. Comparing the Raman spectrum of the graphite, the graphene, and the GNSs shown in Fig. 3a, we can see that the structure of the GNSs is more similar to the bulk graphite rather than to the graphene sheets except that their frequency and intensity of the characteristic peaks are a little different. The ratio of G peak (~1580 cm-1) to 2D peak (~2700 cm^−1^) and the shift of the 2D peak are often used to judge the layer number^22^. Accordingly, the graphene sheets which scrolled to the GNSs are a few layer numbers which are agreement with the results characterized by TEM and AFM shown in Fig. S1. The interlayer spacing of the GNSs is also calculated to be around 0.34 nm according to the value of 2θ of the (002) peaks of the XRD patterns of the GNSs (Fig. 3b), which matches well with the TEM and Raman spectrum. Moreover, we have proved that the nanoparticles on the surface of the GNSs shown in Fig. 2d are AgCN by comparing the diffraction characteristic peaks of the GNS with the PDF card of AgCN as shown in Fig. 3b. The detail demonstration work has been reported in the recently published article^25^.

**Figure 4:**
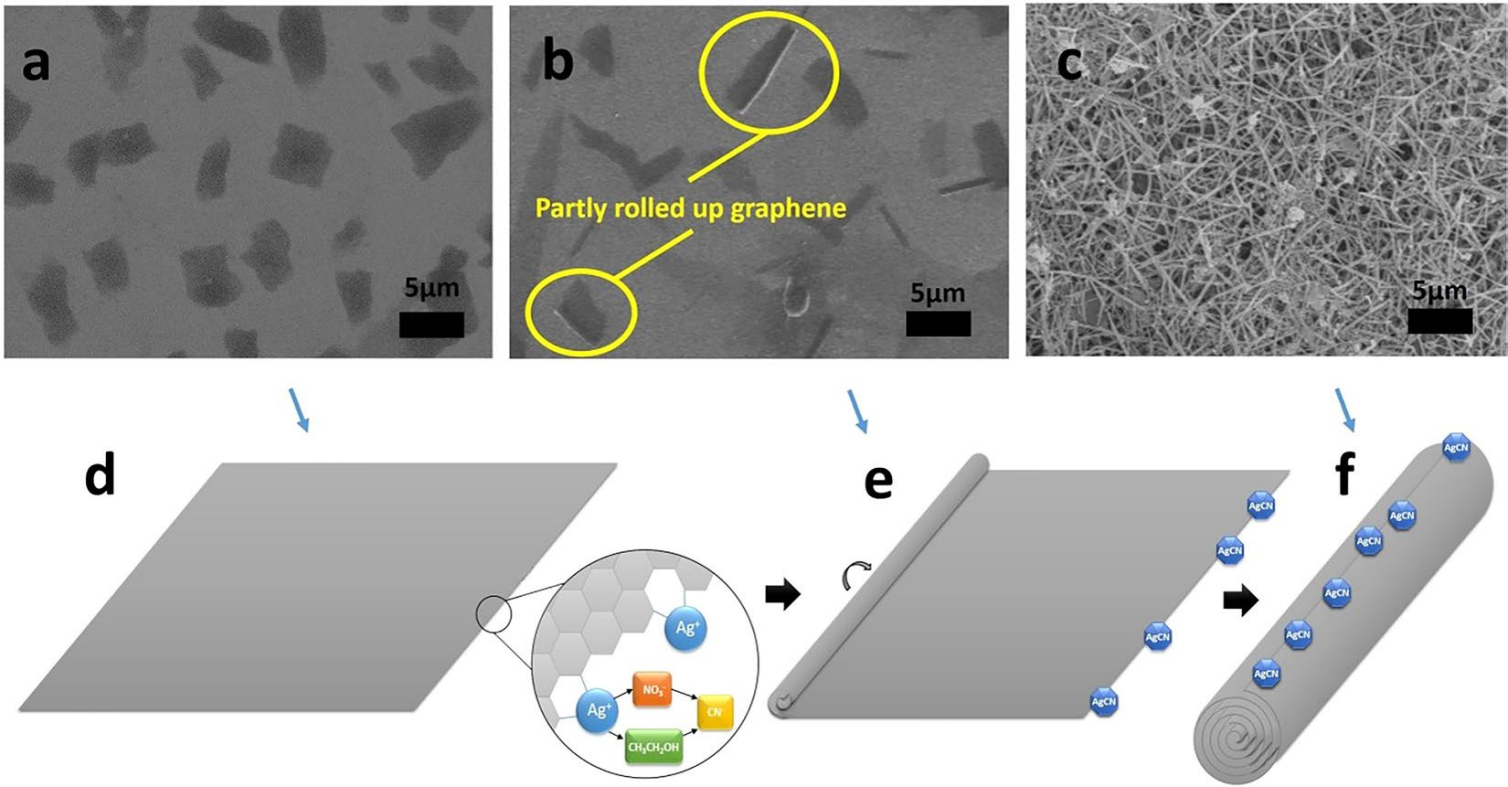
SEM images of the graphene (**a**), intermediate transition state (**b**) and the converted GNSs (**c**). The speculation mechanism of the scrolling process of the graphene into the GNSs. A square graphene sheet and the position occupied with the created AgCN (**d**), the partially curled state of the graphene and the formed AgCN particles at the edge (**e**), the final finished GNSs and formed AgCN particles on the surface of the GNSs (**f**).

In order to explore the conversion process of the graphene to the GNSs, we have examined the change of the morphology and structure of the graphene with the reaction period via the SEM characterization. Figure 4a–c illustrates the morphology of the initially exfoliated graphene, the partially-curling graphene, and the GNSs. The profile of the typical exfoliated-graphene sheets before the reaction is displayed in Fig. 4a. It can be seen that most of the layers are rectangle-like and uniform in size. When the reaction went in 10 min, we can see from Fig. 4b that the graphene sheets had been partly rolled up. When the reaction continued to go on for 30 min, all the graphene sheets had been scrolled up into the GNSs (Fig. 4c). The curling process of the graphene into the GNSs can be explained in the way as shown in the Fig. 4d–f. The AgCN nanoparticles generated at the edge of the graphene play a crucial role in the formation of the GNSs. Initially, the AgCN was created at the edge of the graphene because of the activated dangling bonds of the graphene when the ethanol solution of the graphene was mixed with the ethanol solution of the silver nitrate. The resulted AgCN would change the electron density on the surface of the graphene affecting the adsorption of ethanol solvent on the graphene, increasing the surface energy of the graphene. Thus, when the surface energy of the graphene accumulated to a high level with the increase of the forming AgCN particles (Fig. 4e), the few-layer graphene sheets were triggered to curl up to reduce its surface energy until the complete-scrolls (GNSs) were formed (Fig. 4f). The newly generated AgCN particles trigger the graphene sheets to scroll up. Once the edge of the end of the graphene sheet overlaps its layer, the scrolling-up process continues to the end because of the van der Waals interactions. At this time, the bending energy of the graphene is offset by the der Waals interactions. This speculation is proved by the position of the AgCN particles on the GNSs, all of which are found to be at the exposed edge of the GNSs (Figs 2d and S2). Also, the speculation can be proved by comparing the real size of the GNSs measured from the SEM and TEM images with that calculated based on the mechanism. Suppose a graphene sheet is a square shape and the rolling way is layer-by-layer self-assembly (Fig. S3), the diameter of the GNSs can be calculated to be about 15 nm and 66 nm based on the length of the GNSs ranging from 0.5 µm to 10 µm. The calculation results (the relationship between the length and the diameter of the GNSs) are in good agreement with that made from the SEM and TEM images (Fig. 2a–d). It suggests that the mechanism of the forming GNSs aforementioned is reasonable. It is worth to mention that the GNSs should have superior electronic property because the electron conduction inside the GNSs is on the same graphene surface.

**Figure 5:**
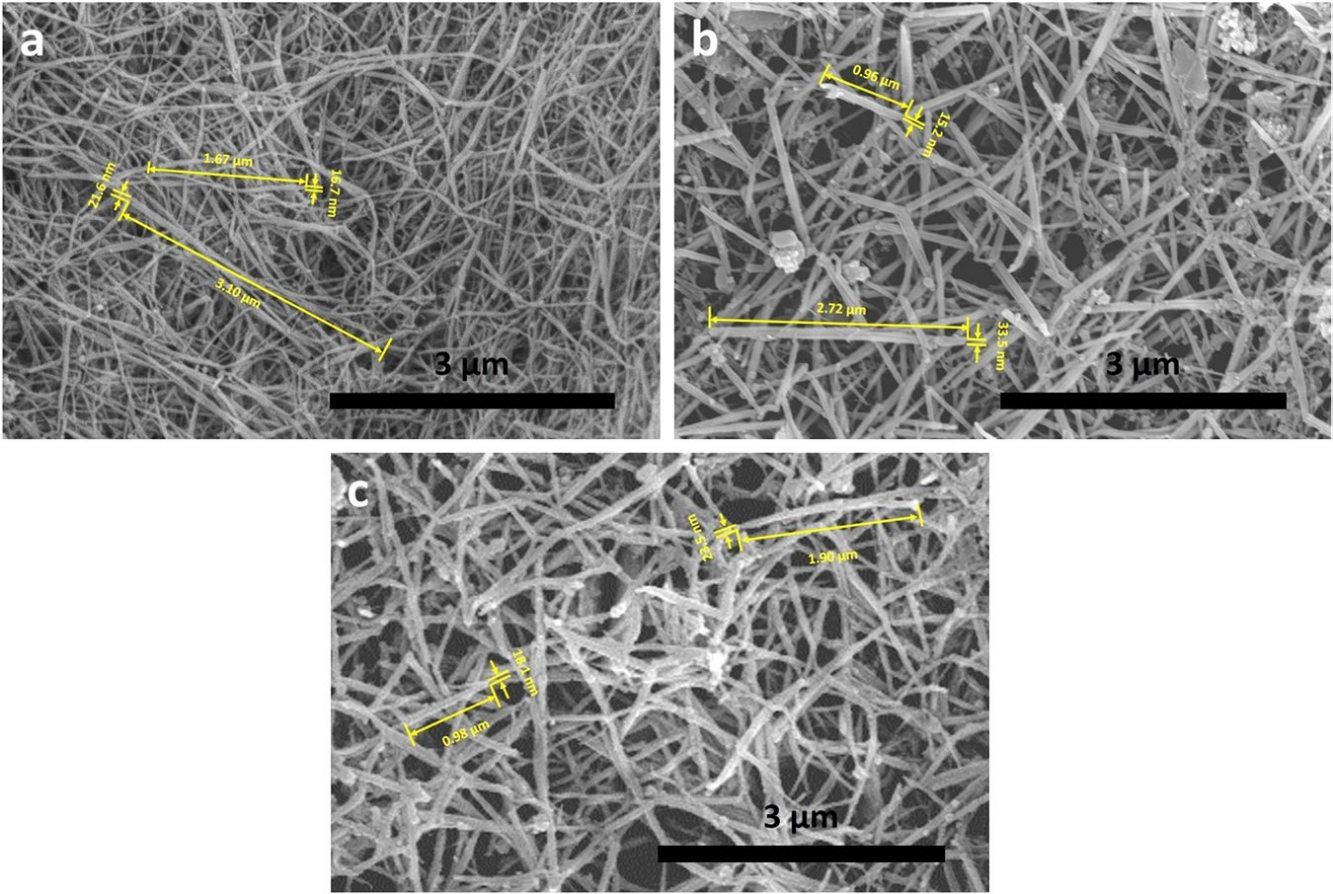
Micrograph of the NSs. SEM images of (**a**) the NSs of h-BN, (**b**) the NSs of MoS_2_ and (**c**) the NSs of WS_2_.

We extend this approach to make other 2DNMs such as h-BN, MoS_2,_ and WS_2_ to explore its generality. The SEM images (Fig. 5a–c) indicated that the exfoliated 2DNMs had been converted into the nanoscrolls (denoted NSs) with a high yield. The Raman spectrum of the bulk h-BN, the bulk MoS_2_, the bulk WS_2_, their initially exfoliated nanosheets, and the NSs indicate that these bulk 2DNMs were peeled off into a few-layer number and in turn converted into the NSs (see Section 4 in supplementary materials). In the similar way of forming the GNSs, the formed AgCN triggered the 2DNMs to transform into the NSs. The AgCN nanoparticles on the NSs were confirmed from the XRD patterns of the NSs of the h-BN, MoS_2_, and WS_2_ (see Section 4 in supplementary materials). Also, we investigated the influence of the mass ratio of 2DNMs and AgNO_3_ on the fabrication of the NSs. As shown in Fig. S5(b,c), only when the mass-ratio between AgNO_3_ and 2DNMs is 0.002 or more, can the NSs be formed. The diameter of the NSs made from the h-BN was calculated to be from 15 nm to 65 nm based on the length varying from 0.5 µm to 10 µm, respectively, which are accorded with the result made via SEM (Fig. 5). The ratio between the length and the diameter is almost as the same as the GNSs. It might be attributed to the fact the h-BN has the same interlayer space as the graphite. Similarly, the relationship between the calculated diameter and the length of the NSs made from the MoS_2_ and WS_2_ are consistent with the results obtained from the SEM. Therefore, these results support the scrolling mechanism aforementioned, and the scrolling process reported in this work is universal.

In conclusion, we have demonstrated a simple and general method to produce high-quality NSs. The single- or few-layer 2DNMs can quickly curl up into the NSs by mixing their ethanol solution with AgNO_3_ ethanol solution at room temperature. It is the AgCN particles generated at the edge of the 2DNMs sheets that trigger the curling process. The successful formation of the NSs from the exfoliated Graphene, h-BN, MoS_2,_ and WS_2_, respectively, substantiates that this approach can be potentially extended to other 2DNMs. Also, the conversion of 2DNMs into their corresponding NSs reaches almost 100%. This work paves the way for largely producing the NSs and carrying out the foundational study and practical application of the NSs.

## Methods

### Chemicals and Reagents

Graphite powder was purchased from Sinopharm Chemical Reagent Co., Ltd (China). H-BN bulk powder was obtained from Strem Chemicals. The MoS_2_ bulk powder was purchased from Alfa Aesar. The WS_2_ bulk powder was purchased from Aladdin. Ethanol (99.7%), AgNO_3_ were purchased from Sinopharm Chemical Reagent Co., Ltd (China). Carbon dioxide (99.9%) was obtained from Shanghai high-tech Co., Ltd (China).

### Exfoliation of 2DNMs

Graphene, h-BN, MoS_2_, and WS_2_ were exfoliated using a shear mixer in a supercritical CO_2_ approach similar to the previously reported paper^26^. The 1 g raw material was put into the reactor, and carbon dioxide was pumped into the reactor by the manual pump. When the pressure and the temperature reached a preset value of 12 MPa and 45 °C, the shear mixer was started and remained at a speed of 3000 r/min for 1 hour. The resultant sample was dispersed by an ultrasonic water bath and centrifuged. More than 80% of the exfoliated 2DNMs were confirmed to be 1 to 5 layers by AFM, TEM and Raman spectra.

### Synthesis of NSs

A certain amount of the exfoliated 2DNMs and AgNO_3_ was dispersed in ethanol solvent in a predefined mass ratio. When the dispersion was magnetically stirred for 30 min at room temperature, the 2DNMs rolled up and deposited as shown in Figs 1 and S6. Then the resulted sediment was separated and dried at 60 °C to obtain the NSs. The as-prepared NSs were stored in the bottle for later characterization.

### Characterization

Scanning electrode microscope imaging was performed using an FEI Nova NanoSEM 450 (USA). Transmission electron microscope images were obtained using JEOL JEM-2100 TEM (Japan) at an acceleration voltage of 120 kV. The X-ray diffraction (XRD) recorded on a BRUKER D8 Advance X-Ray Diffractometer (Germany) using a Cu Kα radiation source (λ = 1.5418 Å). The Raman spectroscopy was recorded on a Raman Microscope excitation (USA) with a wavelength of 532 nm. Zeta Potential was performed with a Malvern Instruments ZS90 Particle Size and Zeta Potential Analyzer (UK).

## Electronic supplementary material

Supplementary Information
