# Supplementary material for: A facile and general approach for production of nanoscrolls with high-yield from two-dimensional nanosheets
Source: Sci Rep. 2018 Oct 15;8:15262. doi: 10.1038/s41598-018-33709-z (PMC6189129; doi:10.1038/s41598-018-33709-z)
Supplement: Supplementary file 1 — Supplementary Information [file 41598_2018_33709_MOESM1_ESM.docx]

Supplementary Materials

A facile and universal approach for massive production of nanoscrolls with high-yield from two-dimensional nanosheets

Wucong Wang, Yanzhe Gai, Ding Xiao, Yaping Zhao*

School of Chemistry and Chemical Engineering, Shanghai Jiao Tong University, Shanghai 200240, P. R. China

*Corresponding author: E-mail:ypzhao@sjtu.edu.cn

1. **The morphology and layer number of the exfoliated graphene sheets**

We applied TEM and AFM to characterize the morphology and the layer number of the exfoliated graphene sheets as shown in Fig.S1. It can be observed from the TEM images of the graphene sheets (Fig. S1a) that the graphene sheets were thin and transparent. Some of them were folded and wrinkled. The high-resolution image (inset) suggests that the layer number was 1-2. It can be seen from AFM image (Fig. S1b and c) that the mean thickness was less than 2 nm suggesting that the layer number of the graphene sheets were less than 5 layers and the lateral size was 1-3 µm.


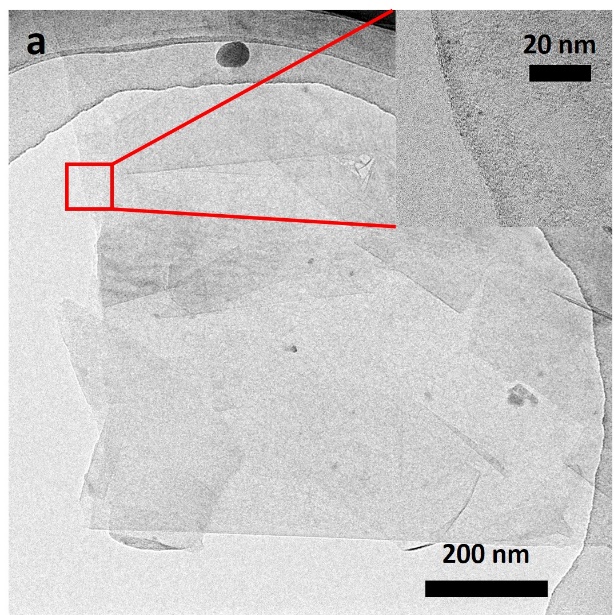

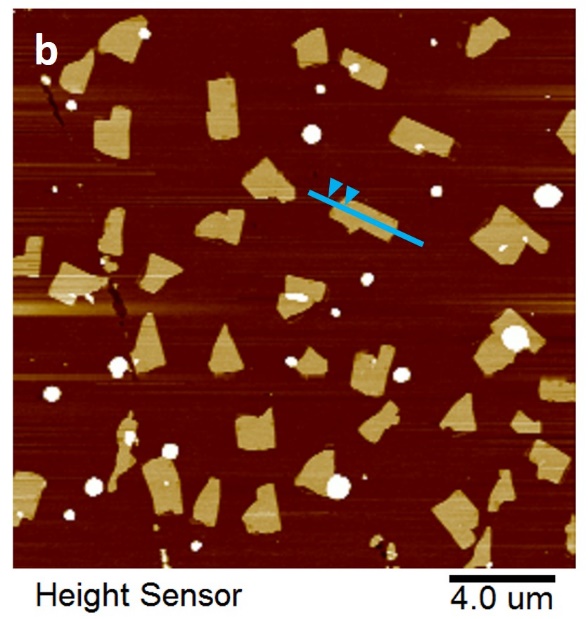


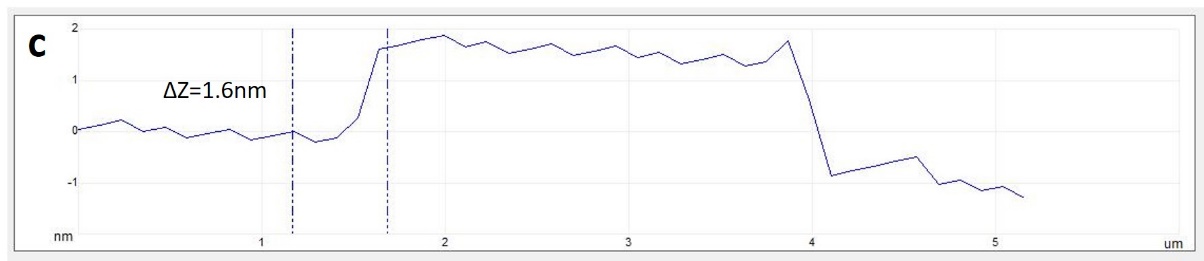


Fig. S1. (a) TEM image and a high-resolution image of the graphene, (b) AFM image of graphene sheets, (c) height profile along the lines shown in panel

1. **Distribution of AgCN**

Fig. S2 indicates that the AgCN particles generated at the edge of 2D nanosheets assembled as a line on the surface of the NSs.


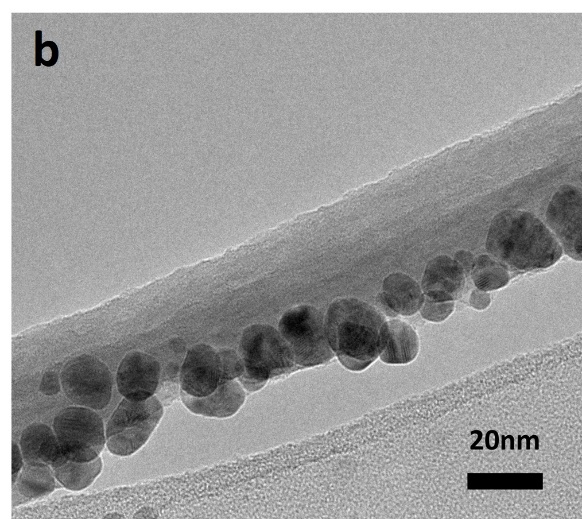


**Fig. S2.** TEM of AgCN nanoparticles on a GNS.

1. **Model for calculation of the size of the NSs**

2D nanosheet (including graphene, h-BN, MoS_2_, and WS_2_) is simplified as a square sheet with side length l and thickness t, as shown in Fig. S3. When the nanosheet rolls up to be a nanoscroll (NSs), the side face of the nanosheet becomes the end section of the nanoscroll. Assuming that, the scroll is free of void, the area of the two surfaces approximately equals each other. The end section of the NSs is simplified as a perfect circle. Then the diameter of the NSs, d, can be estimated using the following equation.

$$lt=\pi\left( \frac{d}{2} \right)^{2}$$

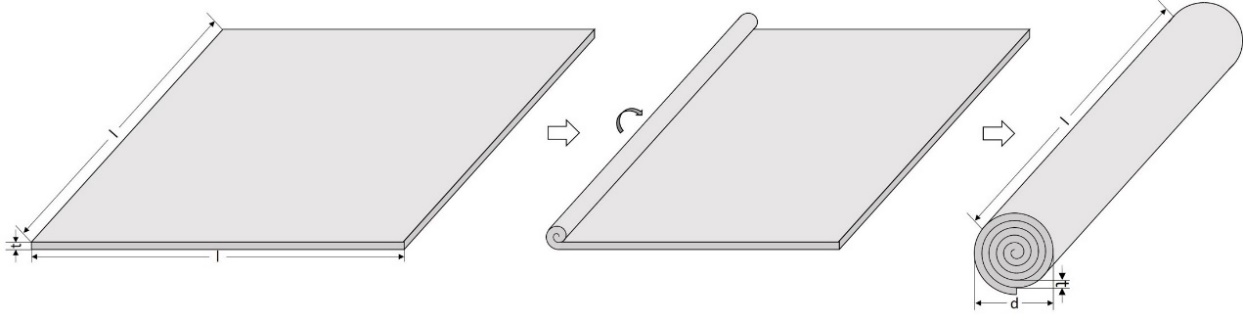


**Fig. S3.** Schematic illustrations of the model of nanoscrolls converted from 2D nanosheet

1. **Characterization of the NSs converted from** few-layer h-BN, MoS_2,_ and WS_2_

The exfoliated few-layer h-BN, MoS_2,_ and WS_2_ were scrolled by the similar method with the graphene. The obtained nanoscrolls (NSs) were characterized by XRD and Raman spectrum.

Fig. S4a shows the XRD of the NSs of the h-BN, MoS_2_, WS_2_ and PDF card of AgCN (JCPDS#23-1404). The (002) peaks are positioned at 26.71°, 14.33°, and 14.31° respectively. By the 2θ of each (002) peaks, we can calculate the interlayer spacing d002 of the NSs made from the h-BN (0.333nm), MoS_2_ (0.617nm) and WS_2_ (0.619nm). It reveals that the interlayer spacing of the NSs was nearly the same with that of their corresponding multilayer structure. This result was the same as the that of GNSs too.

The Raman spectrum of the bulk h-BN, MoS_2_, WS_2_, their corresponding exfoliated nanosheets, and the NSs are shown in Fig. S4 b-d. The E2g phonon characteristic peaks of the bulk h-BN, the exfoliated BN and its NSs (Fig. S3b) were caused by the B–N bond vibration and phonon dispersion within a plane. The peak shows a blue shift from the bulk h-BN (1365 cm-1) to the exfoliated h-BN (1366 cm-1), then a redshift from the exfoliated h-BN to the h-BN NSs (1363 cm^-1^). The blueshift indicates a slightly shorter of the B–N bond caused by the isolated monolayers or compressive stresses. The redshift suggests a small elongation of the B-N bonds created by the scroll structure[^1^](#_ENREF_1). Both MoS_2_ and WS_2_ displays two prominent peaks due to the in-plane (E12g) and out-of-plane (A1g) modes of vibration, respectively (Fig. S4c, d). The frequency of the E12g peak increased, while the frequency of the A1g peak decreased with a decreasing the number of layers. For the MoS_2_ and WS_2_, the number of layers can be unambiguously determined by the decreases in peak spacing between these modes [^2-4^](#_ENREF_2). The Raman spectrum shows that the peak spacing decreased from the bulk samples to the exfoliated samples, and then increased from the exfoliated sample to the rolled samples, which are similar to the graphite case.


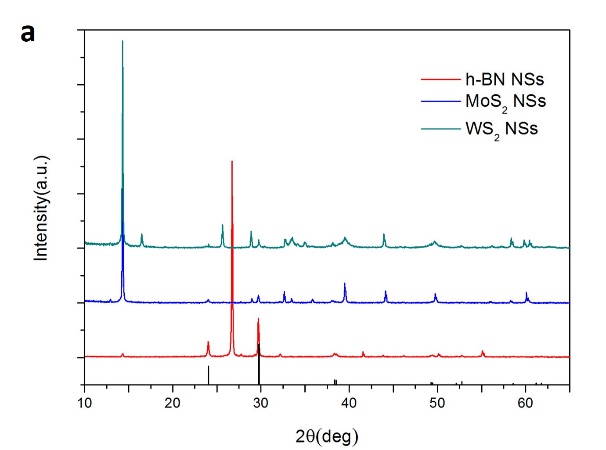

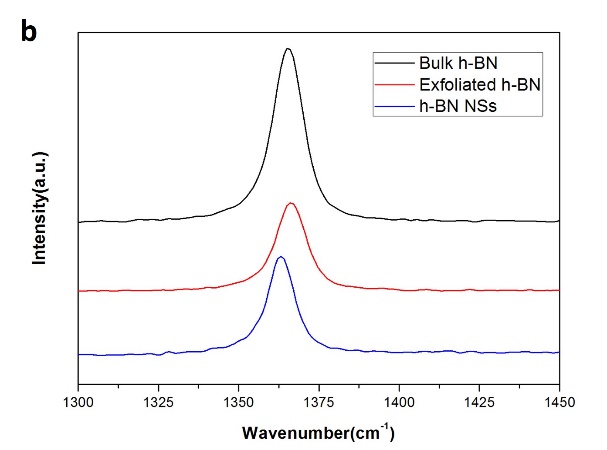


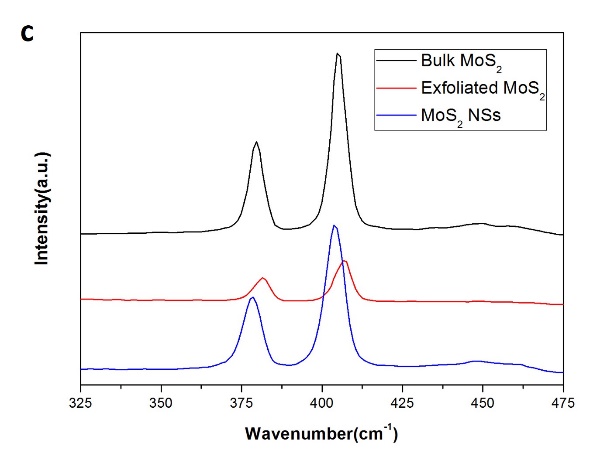

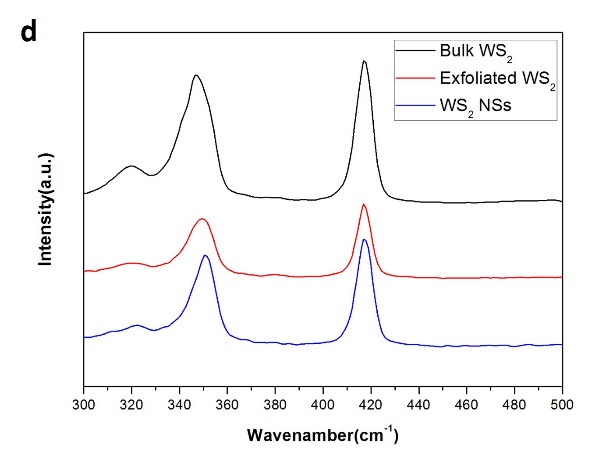


**Fig. S4.** XRD and Raman characterization. (**a**) XRD pattern of h-BN NSs, MoS_2_ NSs, WS_2_ NSs and PDF card of AgCN (JCPDS#23-1404). Observed Raman spectrum of (**b**) bulk h-BN, exfoliated h-BN, h-BN NSs, (**c**) bulk MoS_2_, exfoliated MoS_2_, MoS_2_ NSs and (d) bulk WS_2_, exfoliated WS_2_, WS_2_ NSs.

1. **Digital photos of 2DNMs and 2DNSs**


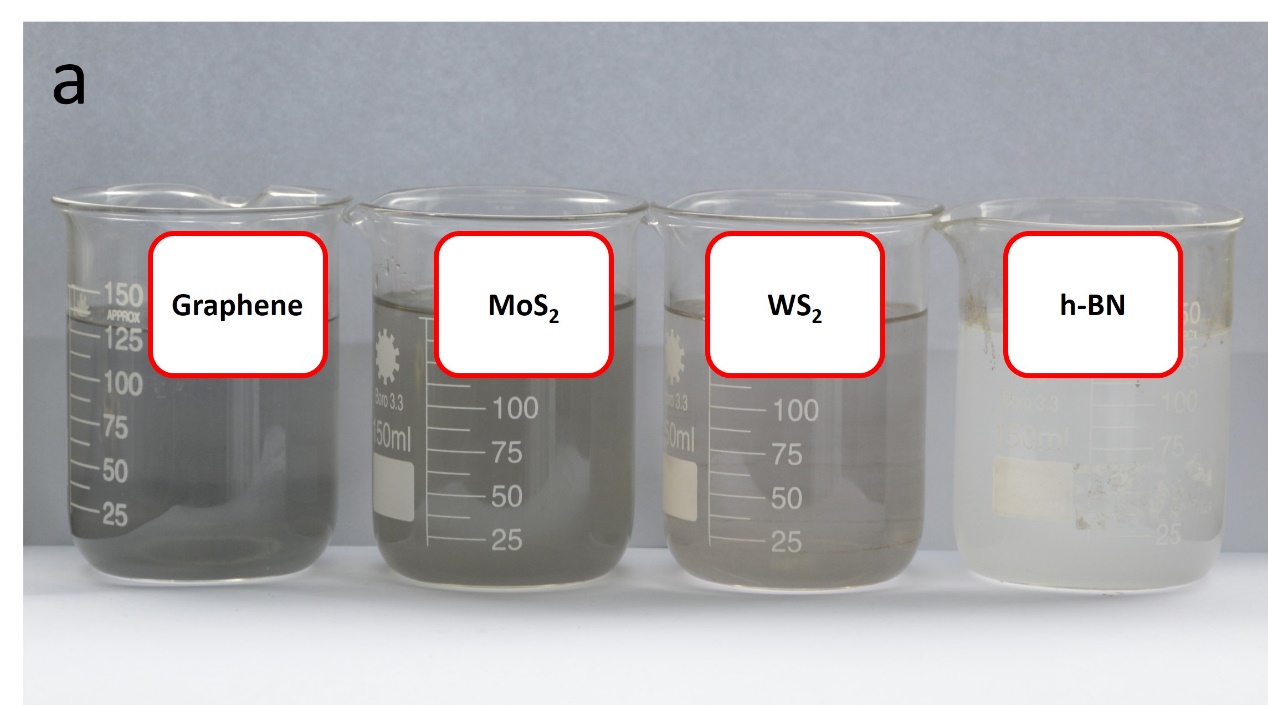


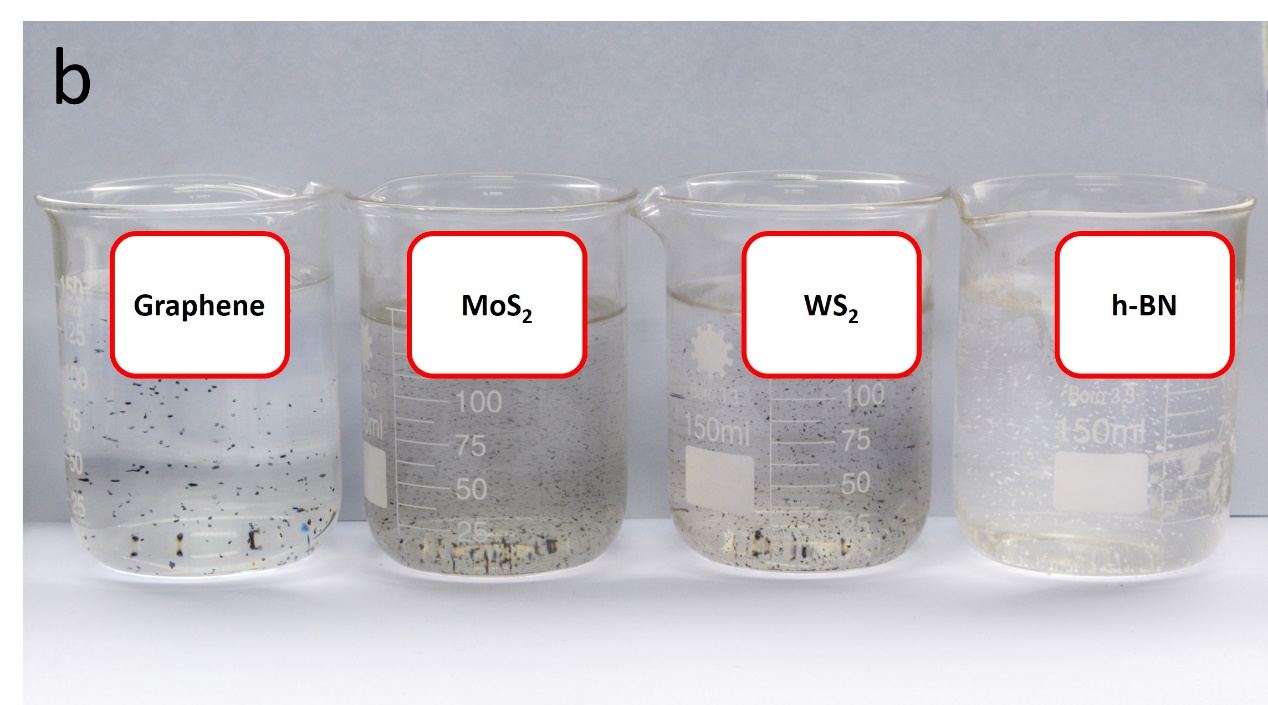


Fig. S5. Digital photographs of (a) ethanol dispersion of the exfoliated Graphene, h-BN, MoS_2_ and WS_2_, and (b) nanoscrolls of the Graphene, h-BN, MoS_2_, and WS_2_.

1. **Influence of the mass ratio of 2DNMs and AgNO_3_ on the formation of scrolls**

The 2DNMs shows a negative Zeta potential in ethanol. When the AgNO_3_ ethanol solution was added dropwise into the 2DNMs ethanol solution, the Zeta potential of the solution increased gradually to positive from the negative value (Fig. S 6a). On this point, the coagulation occurred apparently. Accordingly, we can calculate the mass-ratio of the AgNO_3_ and the 2DNMs to be around 0.01-0.02, on which the 2DNMs were entirely scrolled with an excellent morphological structure. When the mass-ratio of AgNO_3_ and 2DNMs was less than 0.01, the 2DNMs could not be scrolled completely. It might be attributed to the less amount of the AgCN formed. Fig. S6b shows the specifical TEM image of the GNSs made from the mass-ratio of 0.002 between the AgNO_3_ and the GNSs. It can be seen that some graphene sheets were converted into the GNSs. When the mass-ratio was 0.04, the formed AgCN nanoparticles covered almost all the surface of the GNSs as shown in Fig. S6c, which suggests that more AgCN particles were generated. For the h-BN, MoS_2_, WS_2_, we obtained similar results. Therefore, the mass ratio of the AgNO_3_ and the 2DNMs plays an important role in the formation of nanoscrolls.


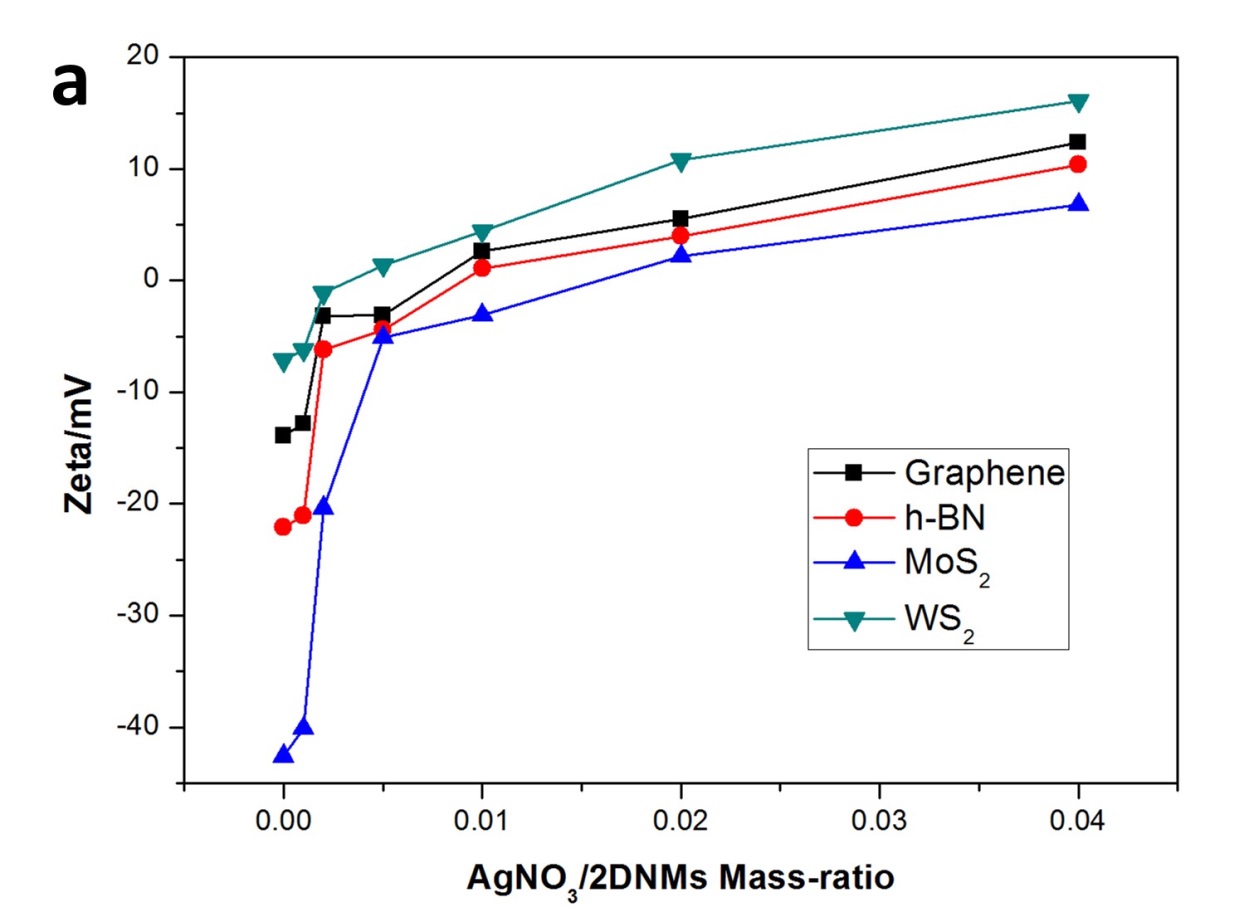


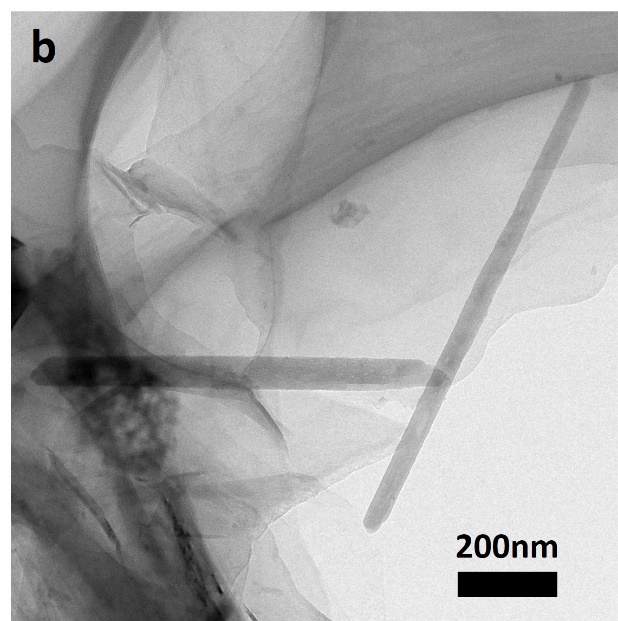

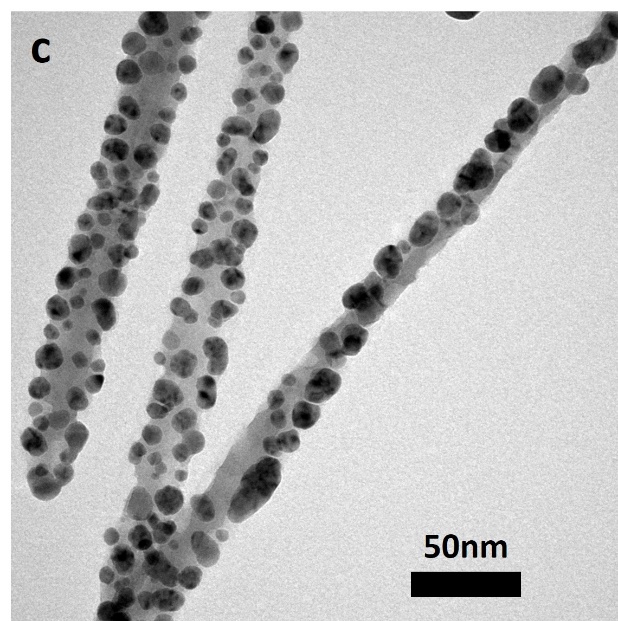


**Fig. S6.** (**a**) Zeta of the mixture by different AgNO_3_ and 2DNMs mass-ratio in ethanol. TEM micrographs of the NSs with the mass-ratio of AgNO_3_ and graphene (**b**) 0.002 and (**c**) 0.04

**Reference**

1 Pakdel, A., Bando, Y. & Golberg, D. Nano boron nitride flatland. *Chemical Society Reviews* **43**, 934 (2014).

2 Zhang, Y. *et al.* Controlled growth of high-quality monolayer WS_2_ layers on sapphire and imaging its grain boundary. *Acs Nano* **7**, 8963-8971 (2013).

3 Meng, J. *et al.* Rolling Up a Monolayer MoS_2_ Sheet. *Small* **12**, 3770-3774 (2016).

4 Thangasamy, P. & Sathish, M. Rapid, one-pot synthesis of luminescent MoS_2_ nanoscrolls using supercritical fluid processing. *Journal of Materials Chemistry C* **4**, 1165-1169 (2016).
